# Molecular diagnosis and genetic diversity of tick-borne Anaplasmataceae agents infecting the African buffalo *Syncerus caffer* from Marromeu Reserve in Mozambique

**DOI:** 10.1186/s13071-016-1715-y

**Published:** 2016-08-17

**Authors:** Rosangela Zacarias Machado, Marta Maria Geraldes Teixeira, Adriana Carlos Rodrigues, Marcos Rogério André, Luiz Ricardo Gonçalves, Jenevaldo Barbosa da Silva, Carlos Lopes Pereira

**Affiliations:** 1 Department of Veterinary Pathology, School of Agrarian and Veterinary Sciences (FCAV/UNESP), Jaboticabal, SP Brazil; 2 Institute of Biomedical Sciences, University of São Paulo (ICB/USP), São Paulo, SP Brazil; 3 National Admnistration of Conservation Areas, Maputo, Mozambique

**Keywords:** African buffalo, *Anaplasma marginale*, *Anaplasma centrale*, *Anaplasma platys*, *Anaplasma phagocytophilum*, Genotyping, *groEL*, *msp5*, *16S* rRNA, Mozambique

## Abstract

**Background:**

Tick-borne diseases (TBDs) are very important in relation to domestic ruminants, but their occurrence among wild ruminants, mainly in the African buffalo *Syncerus caffer*, remains little known.

**Methods:**

Molecular diagnostic methods were applied to detect *Anaplasma marginale*, *Anaplasma centrale*, *Anaplasma phagocytophilum, Ehrlichia ruminantium* and *Ehrlichia chaffeensis* in 97 blood samples of African buffalo captured at the Marromeu Reserve in Mozambique. Molecular detection of agents belonging to the family Anaplasmataceae were based on conventional and qPCR assays based on *msp5*, *groEL*, *16S* rRNA, *msp2*, *pCS20* and *vlpt* genes. Phylogenetic reconstruction of new *Anaplasma* isolates detected in African buffalo was evaluated based on *msp5*, *groEL* and *16S* rRNA genes.

**Results:**

All the animals evaluated were negative for specific PCR assays for *A. phagocytophilum*, *E. ruminantium* and *E. chaffeensis,* but 70 animals were positive for *A. marginale,* showing 2.69 × 10^0^ up to 2.00 × 10^5^*msp1β* copies/μl. This result overcomes the conventional PCR for *A. marginale* based on *msp5* gene that detected only 65 positive samples. Sequencing and phylogenetic analyses were performed for selected positive samples based on the genes *msp5*, *groEL* and *16S* rRNA. Trees inferred using different methods separated the 29 *msp5* sequences from buffalo in two distinct groups, assigned to *A. centrale* and *A. marginale*. The *groEL* sequences determined for African buffalo samples revealed to be more heterogeneous and inferred trees could not assign them to any species of *Anaplasma* despite being more related to *A. marginale* and *A. centrale*. The highly conserved *16S* rRNA gene sequences suggested a close relationship of the new 16 sequences with *A. centrale*/*A. marginale*, *A. platys* and *A. phagocytophilum*.

**Conclusions:**

Our analysis suggests that different species of *Anaplasma* are simultaneously present in the African buffalo. To the best of our knowledge, this is the first study that diagnosed *Anaplasma* spp. in the African buffalo and inferred the taxonomic status of new isolates with different gene sequences. The small fragment of *msp5* sequences revealed to be a good target for phylogenetic positioning of new *Anaplasma* spp. isolates.

## Background

Tick-borne agents (TBAs) form one of the main groups of pathogens infecting both domestic and wild ruminants in sub-Saharan Africa and in tropical and subtropical regions. In Mozambique, theileriosis, ehrlichiosis, anaplasmosis and babesiosis are the most important tick-borne diseases (TBDs), causing significant economic losses to the national cattle industry [1].

Wild ruminants may play a role as hosts and reservoirs for several tick-borne pathogens, especially Anaplasmataceae agents and piroplasms. For instance, *Anaplasma phagocytophilum, Ehrlichia chaffeensis* and *Ehrlichia ruminantium* are of major concern due to their importance in veterinary and/or human medicine. *Anaplasma phagocytophilum* is transmitted by ticks of the genus *Ixodes* and causes tick-borne fever in sheep, goats and cattle in Europe and granulocytic anaplasmosis in humans [2, 3]. This pathogen has been recognized as the causal agent of illnesses in ruminants in Scotland (United Kingdom), Ireland and Scandinavia [4]. *Ehrlichia chaffeensis*, which is transmitted by the tick *Amblyomma americanum* in the USA*,* is the causative agent of human monocytic ehrlichiosis [5]. White-tailed deer (*Odocoileus virginianus*) are considered the natural reservoirs for both pathogens in wildlife in the USA [4]. In turn, *E. ruminantium* has been reported in Africa and can be transmitted by ticks of the genus *Amblyomma* (especially *A. variegatum* and *A. hebraeum*). Currently, ehrlichiosis is considered to be one of the most important diseases of domestic ruminants in sub-Saharan Africa [6], with a high mortality rate among susceptible sheep, goats and cattle. Although the African buffalo is considered to act as wild reservoir for this agent, clinical signs and prevalence in this animal species remain little known [7, 8].

Although several tick-borne agents (TBA) may affect buffalo, special attention needs to be paid to *A. marginale,* since this is an important pathogen that is responsible for significant economic losses relating to cattle-rearing in South America and Africa [9]. In these regions, this bacterium can be transmitted mechanically by hematophagous dipteran insects, including various species of *Tabanus* and *Stomoxys*, and by some mosquito species in the genera *Culex* and *Aedes* [10]. Although *A. marginale* has already been detected in several wild ruminant species, such as *Odocoileus virginianus*, *O. hemionus hemionus*, *O. hemionus columbianus*, *Antilocapra americana*, *Cervus elaphus nelson* and *Ovis canadensis canadensis* in North America and *Connochaetes gnou*, *Damaliscus dorcas phillipsi* and *Sylvicapra grimmia grimmi* in Africa [11], most of the studies on the occurrence, seroepidemiology and molecular characterization of these agents have been conducted among cattle [12, 13]. When *A. marginale* infects ruminant species other than cattle, the infection is generally of a chronic nature [11].

In Mozambique, large numbers of African buffalo are maintained in national parks under the protection of the country’s legislation. However, there are still no studies on the prevalence of tick-borne pathogens circulating in this group of animals. In the present study *Anaplasma* species was detected in African buffalo in the Marromeu Reserve (Mozambique) and characterized based on *msp5*, *groEL* and *16S* rRNA genes for comparison and phylogenetic inferences.

## Methods

### Experimental area

In 2011, blood samples were collected from 97 African buffalo (*Syncerus caffer*) in Mozambique, Marromeu Reserve. This reserve is a special buffalo protection area located in the Marromeu district (Sofala Province), with an area of 1,500 km^2^ (www.jenmansafaris.com). Sampled animals were apparently healthy young male and female individuals. Blood samples had been collected before the animals were transferred from Marromeu Reserve to the Gorongosa Reserve, a distance of *c.*300 kilometers.

### Samples and DNA extraction

Blood samples were collected from the buffalo using EDTA and were mixed (v/v) with ethanol for further DNA extraction. In Brazil, the blood samples from these naturally infected buffalo were incubated in a lysis buffer (1 % SDS, 100 mM EDTA at pH 8.0, 20 mM Tris-HCl at pH 8.0 and 350 mg/ml of proteinase K) at 37 °C for 18 h and centrifuged at 14,000×  *g* for 5 min. The DNA was purified using Wizard Purification Systems (Promega). The concentration of each DNA sample was determined in a NanoDrop 2000c spectrophotometer (Thermo Scientific, San Jose, CA, USA).

### PCR screening for tick-borne pathogens

#### qPCR

A quantitative real-time PCR, based on a fragment of *msp1*β gene of *A. marginale* and previously described by Carelli et al. [14], was used aiming to estimate the parasitemia by means of absolute quantification (number of copies/μl). Additionally, a multiplex qPCR for *A. phagocytophilum* (*msp2* gene) and *E. chaffeensis* (*vlpt* gene) was performed [15]. Serial dilutions of plasmid DNA containing the target sequence were performed aiming to construct standards with different concentrations of the target sequence (2.0 × 10^7^ copies/μl to 2.0 × 10^0^ copies/μl) of studied agents. The number of plasmid copies was determined in accordance with the formula (X g/μl DNA/[plasmid size (bp) × 660]) × 6.022 × 10^23^ × plasmid copies/μl. The amplification reactions were performed using a final total reaction volume of 10 μl, containing a mixture of 1.0 μl of sample DNA, 0.2 μl of probe, 0.9 μl of each primer, 5.0 μl of PCR buffer (IQ Multiplex Power Mix®, BioRad) and 2.0 μl of ultra-pure sterile water (Nuclease-Free Water®, Promega).

#### Nested PCR

DNA samples were screened by different conventional PCR assays: a nested PCR for *E. ruminantium* [16]; two nested PCRs for *Anaplasma* spp. based on partial sequences of the *16S* rRNA gene for detection of *A. phagocytophilum*, *A. bovis* and *A. platys* [17], and for *A. centrale* and *A. marginale* [18]; a nested PCR for the *groEL* gene [19–21]; a PCR based on the major surface protein 5 (MSP5) gene [22, 23]. For these different protocols we followed similar PCR conditions. For the nested PCR, the first reaction was conducted in a final volume of 25 μl of the mixture, containing 5 μl of genomic DNA, 12.5 μl of Taq PCR Master Mix (Qiagen, Madison, USA), 6.5 μl of ultra-pure water and 0.5 μl of each primer. In the second reaction, a final volume of 25 μl of the mixture was used, consisting of 1 μl of the product that had been amplified in the first reaction, 12.5 μl of Taq PCR Master Mix, 10.5 μl of ultra-pure water and 0.5 μl of each primer.

Ultra-pure sterile water was used as negative control in all the PCR assays described above. *Anaplasma phagocytophilum* and *E. chaffeensis* DNA positive controls were kindly provided by Dr. J. Stephen Dumler (University of Maryland, Baltimore, MD, USA). The Jaboticabal strain of *A. marginale* was used as positive control [GenBank accession number KJ398398]. In order to prevent PCR contamination, DNA extraction, reaction setup, PCR amplification and electrophoresis were performed in separate rooms.

### Sequencing and phylogenetic analysis

The samples that amplified PCR products corresponding to *msp5*, *groEL* and *16S* rRNA genes were purified using a Silica bead DNA gel extraction kit (Thermo Scientific, San Jose, CA, USA), in accordance with the manufacturer’s recommendations. The purified material was quantified in a Nanodrop spectrophotometer (Thermo Scientific, San Jose, CA, USA). Sequencing was performed on the purified products using the same set of PCR reaction primers, in an automated sequencer (ABI PRISM 3700 DNA Analyzer; Applied Biosystems, CA, USA) at the Biological Resource and Genomic Engineering Center (CREBIO), FCAV, UNESP, Jaboticabal.

For the phylogenetic analysis on *msp5*, *groEL* and *16S* rRNA gene sequences determined in this study, different *Anaplasma* spp. and related species were used.

Three alignments of partial *msp5* (351 bp), *groEL* (520 bp) and *16S* rRNA (502 bp) gene sequences were constructed using Clustal X [24] and adjusted manually. The phylogenetic analyses were carried out using different methods: the neighbour-joining (NJ) algorithm was run in the Mega 4 software [25]; maximum parsimony (MP) and bootstrap analyses were carried out using PAUP* 4.0b10 [26] with 100 replicates of random addition sequence followed by branch swapping (RAS-TBR), as previously described [27]; and Maximum Likelihood (ML) analyses were performed using RAxML v.2.2.3 [28]. Bayesian Inference (BI) analysis was done using MrBayes on XSEDE (3.2.6) [29] in Cipres Science Gateway [30]. Akaike information criterion was used in Mega 4 [25] to identify the best-fitting model of nucleotide substitution. The phylogenetic analyses using *msp5*, *groEL* and *16S* rRNA genes were performed with GTR + gamma, GTR + gamma + proportion of invariable sites and TN93 + proportion of invariable sites models, respectively. The first 25 % of the trees from 100,000,000 generations were discarded as 'burn-in'.

## Results

### Diagnostic evaluation of *Anaplasma* spp. and *Ehrlichia* spp. in African buffalo blood samples

Different specific PCR assays were used to detect Anaplasmataceae DNA in blood samples from African buffalo in the Gorongosa National Park, Mozambique. All the 97 blood samples tested were negative in a specific nested PCR for *E. ruminantium* (based on *pCS20* gene) [16] and in a multiplex real-time PCR for *E. chaffeensis* (based on *vlpt* gene) and *A. phagocytophilum* (based on *msp2* gene) [15]. Seventy African buffalo (72.2 %) were shown to be positive for amplification of fragments of the gene *msp1*β in the real-time PCR specific for *A. marginale*. Sixty-five African buffalo (67.0 %) were shown to be positive in the semi-nested PCR for *A. marginale* based on a fragment of the gene *msp5*. All of the animals that were positive in the semi-nested PCR were shown to be positive in the qPCR, and five animals that were negative in the conventional PCR were shown to be positive in the qPCR (the mean quantification of these animals was 1.32 × 10^1^ DNA copies/μl of blood). The absolute quantification in the qPCR ranged from 2.69 × 10^0^ to 2.00 × 10^5^ copies of *msp1*β *-A. marginale* DNA per μl of blood.

Out of the 65 samples that were positive for *A. marginale* using msp-5 diagnostic PCR, only 29 samples were selected for sequencing due to higher intensity of bands in agarose gels.

PCR based on *groEL* gene revealed 50 (51.5 %) positive samples for *Anaplasma*; of these, 35 samples were selected for DNA sequencing. We also used two different assays based on *16S* rRNA gene aiming the amplification of DNA for *A. centrale*, *A. marginale*, *A. platys*, *A. bovis* and *A. phagocytoplilum* [17, 18]. We only identified 16 positive samples for *16S* rRNA gene using the protocols described above and all samples were sequenced.

### Genetic variability within *A. centrale* and *A. marginale* clades inferred from *msp5* gene sequences

**Fig. 1:**
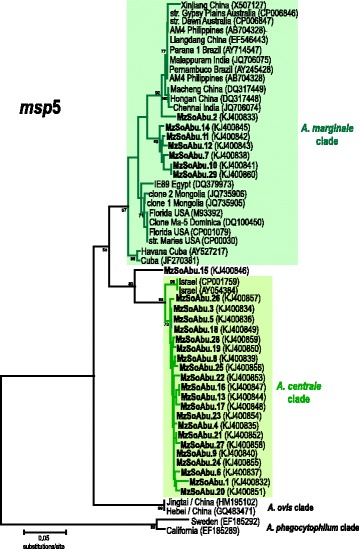
Phylogenetic tree based on the *msp* *5* gene sequences amplified from 29 blood samples of African buffalo and sequences for *Anaplasma* spp. retrieved from the GenBank database. Tree reconstruction was inferred using the Neighbour-Joining method (maximum composite likelihood model; pairwise deletion) with 500 bootstrap replications. *Anaplasma phagocytophilum* was used as the outgroup

In this study, we sequenced partial *msp5* sequences for *Anaplasma* spp. amplified from wild African buffalo blood samples in Mozambique, East Africa. We determined 29 sequences for the *msp5* gene that showed high identity to *Anaplasma* spp. in a BLAST search in the NCBI website (www.ncbi.nlm.nih.gov). The sequences were aligned with all *Anaplasma msp5* gene sequences available in the GenBank database (Fig. 1), including data from the genome project on three samples of *A. marginale* and one sample of *A. centrale*. A set of 18 *A. marginale* and one *A. centrale* sequences from North, Central and South America, Asia and Australia was included in the analysis, with the aim of placing the new sequences in the general phylogeny of *Anaplasma* spp. *Anaplasma phagocytophilum* was used as the outgroup in all of the tree reconstructions.

Analyses performed using different methods (Neighbour Joining, Maximum Parsimony, Maximum Likelihood and Bayesian Inference) yielded similar tree topologies and the same relationships for all of the major groups identified in this study; the NJ tree was chosen to represent the relationship observed (Fig. 1). According to the tree topologies inferred using nucleotide sequences (351 bp), the 29 partial *msp5* sequences were distributed into two main groups, composed of different isolates of *A. centrale* and *A. marginale* of different geographical provenance. These two species are known to be always more related to each other than to *A. ovis*, and the tree topology inferred here was consistent with previous studies [31], although only partial *msp5* sequences (~351 bp) were used in this analysis. Most of the sequences (21 samples) were clustered (with 99 % bootstrap support) with the only two available (and identical) *msp5* sequences for *A. centrale* (genome project strain Israel CP001759 and AY054384) and exhibited 1.49 % of genetic divergence within this group. The positioning and high similarity of *msp5* sequences strongly suggest the identification of these 21 samples as isolates of *A. centrale*. Although this group had the most homogeneous *msp5* sequences, the sample from buffalo MzSoAbu.15 was always positioned at the edge of the group (80 % bootstrap support). This sample showed approximately 8 % genetic divergence from the *A. central*e sequences and 10 % from *A. marginale* sequences, and according to the tree topology was most related to the *A. centrale* isolates.

The largest numbers of *msp5* sequences available in the GenBank database are from *A. marginale* (20 sequences) from isolates with a worldwide distribution (Asia, Australia and South, Central and North America), whose genetic divergences ranged from 0 to 9.8 % (3 % of genetic index of divergences). However, among these 20 *msp5* sequences of *A. marginale*, only the Egyptian sample IE89 (DQ379973) was from Africa. In the present study, a single sample (buffalo MzSoAbu.02) was strongly positioned with *A. marginale* (90 % bootstrap support) and was closely related to isolates of different geographical provenance (North, Central and South America, Asia and Australia). Although positioned together with *A. marginale msp5* sequences, the sample from buffalo MzSoAbu.02 was the most divergent among the newly-generated sequences and showed divergences ranging from 3.7 to 9.8 % with the sequences available for this species. The comparison of *A. marginale msp5* sequences retrieved from GenBank and the genome projects showed divergences ranging from 0 to 7.1 %; the inclusion of African buffalo samples ranged up to ~8.7 %.

The remaining six *msp5* sequences (MzSoAbu.07, MzSoAbu.10, MzSoAbu.11, MzSoAbu.12, MzSoAbu.14 and MzSoAbu.29) always clustered together (89 % bootstrap support and 2.3 % genetic divergence) and diverged by 6.5 % from all *A. marginale* sequences (Fig. 1). When these six sequences were added to the calculation of the genetic index of internal divergence of *A. marginale*, the values changed from 3.0 to 4.0 %. In the current dataset, comprising all the available *msp5* sequences from *Anaplasma* spp., the positioning of these six sequences was always the same, independent of the method used and closeness to *A. marginale*. However, in a reduced dataset, the positioning of these six sequences changed according to the different methods used.

Despite the reduced available data of *msp5* sequences for *A. centrale*, our analysis revealed that there were more heterogeneous sequences of *msp5* among the *A. marginale* sequences (3.0 % internal divergence) than among *A. centrale* sequences (~2.0 % internal divergence, including the newly-generated sequences). In addition, the genetic divergences between *A. marginale* and *A. centrale* groups were clearly evident and supported their distinction into two different clades. *Anaplasma marginale msp5* sequences were separated from *A. centrale* sequences by a genetic divergence of 14 %, from *A. ovis* by 17 % and from *A. phagocytophilum* by 35 %. The few available *msp5* sequences for other *Anaplasma* species were for *A. ovis* (two identical sequences from China) and for *A. phagocytophilum* (two sequences from North America and Europe diverging by 4.4 %).

### Polymorphism of *groEL* sequences close related to *A. centrale* and *A. marginale*

**Fig. 2:**
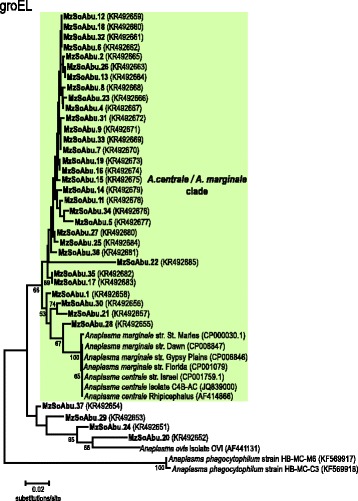
Phylogenetic relationships of *Anaplasma* spp. identified in African buffalo blood samples in Marromeu reserve at Mozambique based on the *groEL* gene. The tree was reconstructed by the Neighbour-Joining method (maximum composite likelihood model; pairwise deletion) with 500 bootstrap replications. *Anaplasma phagocytophilum* was used as the outgroup; the newly-generated sequences are indicated in bold

We generated 35 partial *groEL* nucleotide sequences (~520 bp) and inferences were made using strains of *A. marginale*, *A. centrale*, *A. ovis* and *A. phagocytophilum* for comparative purposes. The aligned region comprised positions 18 to 537 in the sequence for *A. marginale* St Dawn (CP006847) that was used as guide sequence for *groEL* gene. *Anaplasma centrale* and *A. marginale groEL* sequences retrieved from GenBank showed to be identical in the 520 aligned nucleotide positions, with the exception of *A. marginale* St Maries from USA (0.19 %) and *A. centrale* from *Rhipicephalus simus* from South Africa (0.38 %) that diverged from each other by 0.58 %. The high similarity of *groEL* sequences between *A. centrale* and *A. marginale*, two recognized distinct *Anaplasma* species, precluded their separation in the phylogenetic trees inferred (Fig. 2).

Our analysis revealed four heterogeneous sequences from buffalo samples (MzSoAbu.37, MzSoAbu.29, MzSoAbu.24 and MzSoAbu.20) that diverged from each other by 4.0–7.7 % . *Anaplasma ovis* was distantly related to *A. marginale* and *A. centrale* (distance of ~20 %). The above mentioned buffalo samples presented divergence values more closely related to other African buffalo isolates (2–11 %) than to *A. ovis* (6.6–12.3 %). The high degree of similarity presented by* groEL* gene sequences associated with the intriguing positioning and the low bootstrap support preclude associating these sequences with neither *A. ovis* nor *A. centrale/A.marginale* isolates.

Sequences from buffalo samples MzSoAbu.01, MzSoAbu.30, MzSoAbu.21 and MzSoAbu.28 (index of 4 %) showed to be intimately related to the cluster that grouped the clades *A. marginale* and *A. centrale* (divergence ranging between 2–3 %). All of the remaining 27 sequences showed ~3 % of divergence index (ranging from 0.7 to 10 %). The inferences gathered using the *groEL* sequences retrieved from 35 buffalo samples suggest a closer association to *A. centrale/A. marginale* clade than to other species but makes it difficult to infer the degree of relatedness within the reference strains due to high sequence similarity of the *groEL* gene. Our analysis showed a high degree of heterogeneity among and within *Anaplasma* samples evaluated herein (Fig. 2). In addition, we did not observe a congruent phylogeny with inferences using *msp5* gene sequences suggesting that African buffalo may harbor a complex of different strains and/or genotypes of *Anaplasma* species that we could not detect altogether with *msp5* and *groEL* genes.

### Analysis of *16S* rRNA gene sequences discloses African buffalo infection with *A. centrale/A. marginale, A. platys* and *A. phagocytophylum*

We amplified the *16S* rRNA gene sequences from 16 blood samples from African buffalo using different protocols enabling detection of different species of *Anaplasma* and *Ehrlichia* [17, 18]. Identity matches after a Blast search revealed five samples similar to *A. marginale* and *A. centrale* (99 % similarity), ten to *A. platys* (98–100 % similarity) and one to *A. phagocytophilum* (100 % similarity). Phylogenies were inferred using both groups of sequences from different protocols and methods of analysis (MP, NJ, ML and BI). All methods employed for inferring *16S* rRNA gene trees revealed similar tree tolopogies, with the exception of the Bayesian inference analysis that positioned MzSoAbu.12, MzSoAbu.16 and MzSoAbu.27 in *A. centrale*/*A. marginale* clade instead in *A. platys* clade. Three major clades were identified that included sequences from four *Anaplasma* species: *A. phagocytophilum*, *A. platys*, *A. centrale* and *A. marginale*. Despite the negative results obtained in qPCR specific PCR assay based on *msp2* gene, we identified as *A. phagocytophilum* the sequence from buffalo MzSoAbu.20 that was identical with *A. phagocytophilum* group. Sequences for *A. platy*s were related to *A. phagocytophilum* (genetic divergences of ​0.8 %). A total of seven sequences of *Anaplasma* spp. from African buffalo (MzSoAbu.02, MzSoAbu.04, MzSoAbu.10, MzSoAbu.57, MzSoAbu.93, MzSoAbu.80 and MzSoAbu.95) were positioned with *A. platys* and two different uncultured *Anaplasma* spp. (KF010833, KJ831219). A general index of 1 % of genetic divergence was observed in this group and due to the high sequence similarities, some discrepancies were present but showed a better support in the Bayesian inference analysis.

**Fig. 3:**
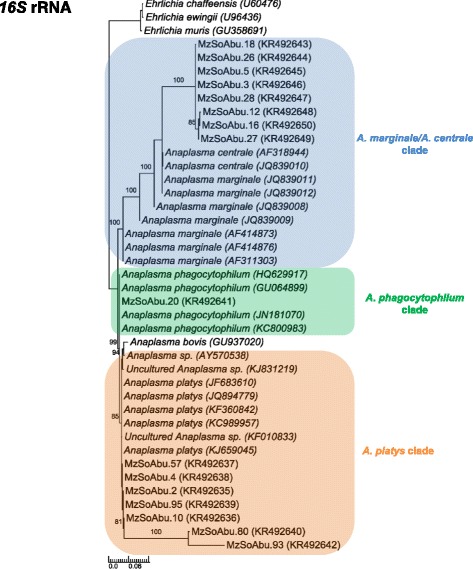
Phylogenetic relationships of *Anaplasma* spp. identified in African buffalo blood samples in Marromeu reserve at Mozambique based on the *16S* rRNA gene. The tree was reconstructed by Bayesian Inference method using MrBayes [number of generations = 100,000,000; MCMC (Markov Chain Monte Carlo) number chains = 4; 'burn-in' = 25 %]. The numbers at the nodes correspond to posterior probability values. *Ehrlichia* spp. were used as outgroups; the newly-generated sequences are indicated in bold

*Anaplasma marginale* and *A. centrale16S* rRNA reference sequences were separated only by 0.2 % and were closely related (0.24 %) to eight sequences from African buffaloes (MzSoAbu.03, MzSoAbu.05, MzSoAbu.18, MzSoAbu.26 and MzSoAbu.28, MzSoAbu.12, MzSoAbu.16 and MzSoAbu.27) that clustered with both *Anaplasma* species (Fig. 3).

The *16S* rRNA sequences generated in this study and used for Blast search identities presented ~500 nt but matched only in 154 nucleotide positions in the alignment due to the specificity of protocols for each group of species (*A. centrale*/*A. marginale* and *A. platys*/*A. phagocytophilum*). The lack of longer sequences to overlap the ~500 nucleotides determined in this study and the high degree of genetic conservation of the *16S* rRNA gene sequences may generate weak and unstable tree topologies. In addition, comparison of all sequences available for *Anaplasma16S* rRNA gene becomes impossible, unless protocols targeting larger fragments have been used for inferences. In this case, the sensibility of PCR assays might be affected and would not detect efficiently the positive samples. In our study, we allied the specificity of PCR protocols to its use for phylogenetic inferences.

## Discussion

The herd of African buffaloes evaluated in this study presented high molecular prevalence of *A. marginale* and, in some cases, the copy numbers detected in the absolute quantification of *msp1*β gene fragment was similar to values observed in cattle with acute infection [14]. It is likely that the involvement of other forms of transmission and the capacity to cause persistent infection [10, 13] make occurrences of *A. marginale* different from those of the other tick-borne agents.

None of the African buffalo was shown to be positive for *A. phagocytophilum*, *E. chaffeensis* and *E. ruminantium*. However, one sequence showing 0.6 % of divergence with *A. phagocytophilum* was obtained using a nested PCR targeting the *16S* rRNA gene, despite the negative results obtained in a specific qPCR PCR based on *msp2* gene.

On the other hand, the high prevalence and high numbers of copies of *A. marginale* per μl observed in the present study suggest that African buffalo are likely to be infected but also possibly present persistent infection and high levels of parasitemia. *Anaplasma marginale* presents worldwide distribution on cattle and is the primary cause of economic losses in cattle herds in developing countries [31]. The prevalence of *A. marginale* found in the present study was higher than the 5.4 % (27/500) and 17 % (20/116) observed among water buffalo in Brazil [32] and Pakistan [33], respectively, but was similar to that found among cattle (79.2 %) in Mozambique [1]. Even in buffalo presenting low levels of infestation by ticks [34] and high immunological competence [35], *A. marginale* presented high prevalence under the conditions studied, thus suggesting that this agent might be transmitted by other hematophagous insects that have not yet been identified, which would thus allow high prevalence to become established, even in rural animals.

Data relating to the vector competence and impact of the transmission of *A. marginale* by hematophagous dipterous insects are still scarce. *Anaplasma marginale* may be transmitted mechanically by different species of tabanids, e.g. *Stomoxys calcitrans*, and some species of mosquitoes, such as those in the genera *Culex* and *Aedes* [10, 36]. However, even though tsetse flies (*Glossina* spp.) are common on the African continent and act as competent vectors for important pathogens such as *Trypanosoma brucei* and *Trypanosoma vivax*, no studies proving the role of these flies in transmitting *A. marginale* in Africa have been conducted yet. According to Scoles et al. [10], mechanical transmission of *A. marginale* depends on the presence of high levels of parasitemia at the time of the blood meal and is thus limited to herds in which animals are in acute phase of infection. The levels of parasitemia assessed by qPCR absolute quantification (ranging from 2.69 × 10^0^ to 2.00 × 10^5^ copies of *msp1ß*-*A. marginale* DNA per μl of blood) presented by African buffalo in Mozambique may facilitate the occurrence of mechanical transmission. Thus, flies may play a role in the transmission of *A. marginale*, given that African buffalo inhabit bush and/or flooded areas to which *Rhipicephalus* spp. ticks are often poorly adapted. In addition, buffalo may act as wild reservoirs for *A. marginale*, which may become a problem for cattle herds that are kept in areas close to National Reserve areas, given that when cattle come into contact with ticks and flies coming from these buffalo, they may develop clinical disease.

Phylogenies inferred in this study based on *msp5*, *groEL* and *16S* rRNA gene sequences did not show congruent tree topologies. The 29 *Anaplasma* spp. *msp5* sequences were identified as *A. centrale* and *A. marginale* with a high confidence of bootstrap support and sequence identities. The phylogeny inferred using 35 new *Anaplasma* spp. *groEL* sequences did not show congruent topology with those inferred using the *16S* rRNA and/or *msp5* genes from this study. A close association to *A. centrale*/*A. marginale* clade was observed for the new 35 *groEL* sequences but the dataset used in this study did not enable  distinguishing even the reference strains from genome projects of both species. Larger fragments or complete gene sequences would help to separate these closely related *Anaplasma* species. However, the *groEL* sequences from African buffalo exhibited heterogeneity thus reinforcing the idea that different genotypes and/or species related to *Anaplasma* circulate among wild animals.

Despite being also conserved, the *16S* rRNA gene sequences positioned the African buffalo samples in three distinct clades: *A. platys* (7 samples), *A. centrale*/*A. marginale* (8 samples) and *A. phagocytophilum* (1 sample). The high specificity of each protocol of *16S* rRNA gene used for diagnosis may not contribute for phylogenetic purposes due to poor data set for alignment construction.

Many of the available *msp5* sequences used in phylogenetic reconstruction dataset were from *A. marginale* isolated from a broad range of localities and hosts (domestic and wild ruminants). The phylogenetic relationships among *Anaplasma* spp. *msp5* sequences inferred in the present study were consistent with the major clades evidenced in previous phylogenies based on *16S* rRNA, *groEL* and gltA gene datasets [31].

Very little is known about the circulating species of *Anaplasma* in wild ruminant hosts in Africa. Considering the divergence among different species and the sequences generated in this study, we can conclude that African buffalo harbor a complex set of genotypes and/or isolates positioned within the *Anaplasma centrale* and *A. marginale* groups. In addition to the lack of sequences for some target genes and poor sampling of *Anaplasma* isolates from different regions, our analysis suggests that protocols for a generic comparison of different genes are needed for a better understanding of tick-borne pathogens circulating in wild animals. The small fragment of *msp5* sequences (345 bp) was helpful in the identification and phylogenetic positioning of new isolates but need to be reappraised for generic comparative purposes.

Phylogenetic inferences based on the gene *msp5* have shown marked similarity between samples of *A. marginale* isolated from cattle and *Riphicephalus microplus* [31]. Our study using *msp5* gene sequences showed that there is high complexity and distinct genotypes, as yet undescribed, within the *Anaplasma centrale* and *A. marginale* circulating in African buffalo. The real impact of these genotypes and/or *Anaplasma* species on the health of buffalo and cattle in Africa requires further study. There are still insufficient data to prove whether the samples of *A. marginale* circulating in African buffalo are transmitted by biological or mechanical vectors.

A detailed understanding of the genetic diversity and phylogenies of *Anaplasma* species from different hosts and geographic regions are still required for elucidating the taxonomic and phylogenetic relationships among the currently recognized *Anaplasma* species, as well as their relationships with other allied species.

## Conclusions

To our knowledge, this is the first molecular study to characterize *Anaplasma* spp. from African buffalo. We detected a high prevalence of *A. marginale* by means of specific qPCR. A *16S* rDNA nested PCR helped to reveal *Anaplasma* species such as *A. phagocytophilum*, *A. centrale*, *A. marginale* and *A. platys* but could not render robust and invariable tree topologies for the closest species. The knowledge of new and genetically complex isolates and/or genotypes from *Anaplasma* species circulating in African buffalo was evaluated with different molecular markers. From our data, a wide range of genetic diversity was recognized among and within *Anaplasma* species in a very ancient group of wild artiodactyl hosts.
